# Sex-Specific Differences in the Effect of Free Testosterone on Sarcopenia Components in Older Adults

**DOI:** 10.3389/fendo.2021.695614

**Published:** 2021-09-22

**Authors:** Hyung Eun Shin, Jeremy D. Walston, Miji Kim, Chang Won Won

**Affiliations:** ^1^ Department of Biomedical Science and Technology, Graduate School, Kyung Hee University, Seoul, South Korea; ^2^ Division of Geriatric Medicine and Gerontology, Department of Medicine, School of Medicine, Johns Hopkins University, Baltimore, MD, United States; ^3^ Elderly Frailty Research Center, Department of Family Medicine, College of Medicine, Kyung Hee University, Seoul, South Korea; ^4^ Department of Biomedical Science and Technology, College of Medicine, East-West Medical Research Institute, Kyung Hee University, Seoul, South Korea

**Keywords:** free testosterone, sarcopenia, community-dwelling older adults, sex-specific difference, aging

## Abstract

**Objective:**

The association of free testosterone (FT) with sarcopenia and its components is well known in men but incompletely understood in women. We examined the association of baseline FT with the prevalence and incidence of sarcopenia and its components in community-dwelling older adults.

**Design:**

Cross-sectional and longitudinal analysis from the prospective population-based Korean Frailty and Aging Cohort Study.

**Methods:**

A total of 1,879 community-dwelling older adults aged 70–84 years were enrolled for cross-sectional analysis and 1,583 subjects who participated in the 2-year follow-up survey were included for longitudinal analysis. Baseline FT levels was measured by radioimmunoassay. Skeletal muscle mass, handgrip strength, and physical performance tests were measured at baseline and after 2-year follow-up. Sarcopenia was defined by the diagnostic criteria of the Asian Working Group for Sarcopenia (AWGS).

**Results:**

Continuous FT levels was positively associated with the prevalence of sarcopenia in men (odds ratio [OR]=0.95; 95% confidence interval [CI]=0.89–1.00)] and women (OR=0.64, 95% CI=0.42–0.99) after adjusting for multiple confounders. In prospective analysis, low FT levels was associated with a decrease in handgrip strength in women (β=-0.61; p=0.010) and a reduction in Timed “Up and Go” (TUG) test (β=0.53; p=0.008) in men after 2 years. No significant correlations were found between FT levels and the incidence of sarcopenia.

**Conclusions:**

Low levels of FT may be a significant determinant of decreases in muscle strength in women and declines in physical performance in men after 2 years. Low FT do not predict loss of muscle mass in both men and women.

## 1 Introduction

Sarcopenia, or the loss of skeletal muscle mass and strength with age, accelerates the risk of adverse health outcomes, such as functional impairment, falls, disability, and mortality in older adults (1, 2). As the population of older adults increases, there have been great efforts to develop methods to counteract the adverse effects of sarcopenia (3). It is essential to identify the risk factors for sarcopenia in order to predict and prevent its development. The etiology of sarcopenia is complex and multifactorial, and includes factors such as advancing age, poor nutritional status, inactivity, pro-inflammatory state, oxidative stress, insulin resistance, and hormonal changes (4–6). Of the hormonal changes, growth hormone, DHEA-S and loss of sex hormones, including testosterone, have all been implicated (7–9).

Testosterone, which is an anabolic steroid hormone, helps maintain muscle mass through influencing satellite cell proliferation and myonuclear number (10, 11). Approximately 70% of testosterone tightly binds to sex hormone-binding globulin (SHBG), and most of the remaining testosterone is bound to albumin. Only about 1% of testosterone in women and 2% in men are in the free form (12). Free testosterone (FT) is a physiologically active fraction of testosterone that is available for tissues (13). Thus, measurement of FT levels can provide a better estimate of biological activities of testosterone.

Serum FT levels progressively decline with age (14, 15). The age-related decline in FT levels has been found to be associated with a reduction in skeletal muscle mass in older men and women (16, 17) and with decreased muscle strength (18) and physical function (19) in older men. Krasnoff et al. (19) demonstrated that baseline FT level was related to the development of mobility limitation in community-dwelling older men after 6.62 years of follow-up. Another study showed the association between low FT level and decreased muscle mass in men after 10 years of follow-up (17). On the contrary, only a couple of studies have reported the association of FT with muscle strength and physical function in older women in cross-sectional analyses. Van et al. demonstrated that the calculated FT levels were positively associated with lean body mass and maximum quadriceps extension strength (20). In addition, Hakkinen et al. found positive correlations between testosterone/SHBG ratio (free androgen index) and maximal force production of leg extensors (21). Testosterone is known to have physiological actions on the body either directly or *via* aromatization to estradiol in women (22). The role of testosterone on the components of sarcopenia remains uncertain in older women because of insufficient clinical data (23). This may be due to difficulties in measuring the very low FT levels in postmenopausal women, although the ovaries continue to secrete androgens in the post-menopause period (24). Furthermore, a previous study found that a disparity in the association between FT level and frailty in older men and women, which suggest that different biological mechanisms may be involved (25). Considering that sarcopenia and frailty share common features, it is possible that the relationship between FT levels and sarcopenia may also show sex-specific difference.

The aim of this study is to examine the association of baseline FT levels with prevalence and incidence of sarcopenia and its components in community-dwelling older adults using a cross-sectional and longitudinal design. We sought to evaluate sex-specific differences in the association of baseline FT levels with sarcopenia and its components.

## 2 Materials and Methods

### 2.1 Study Population

**Figure 1:**
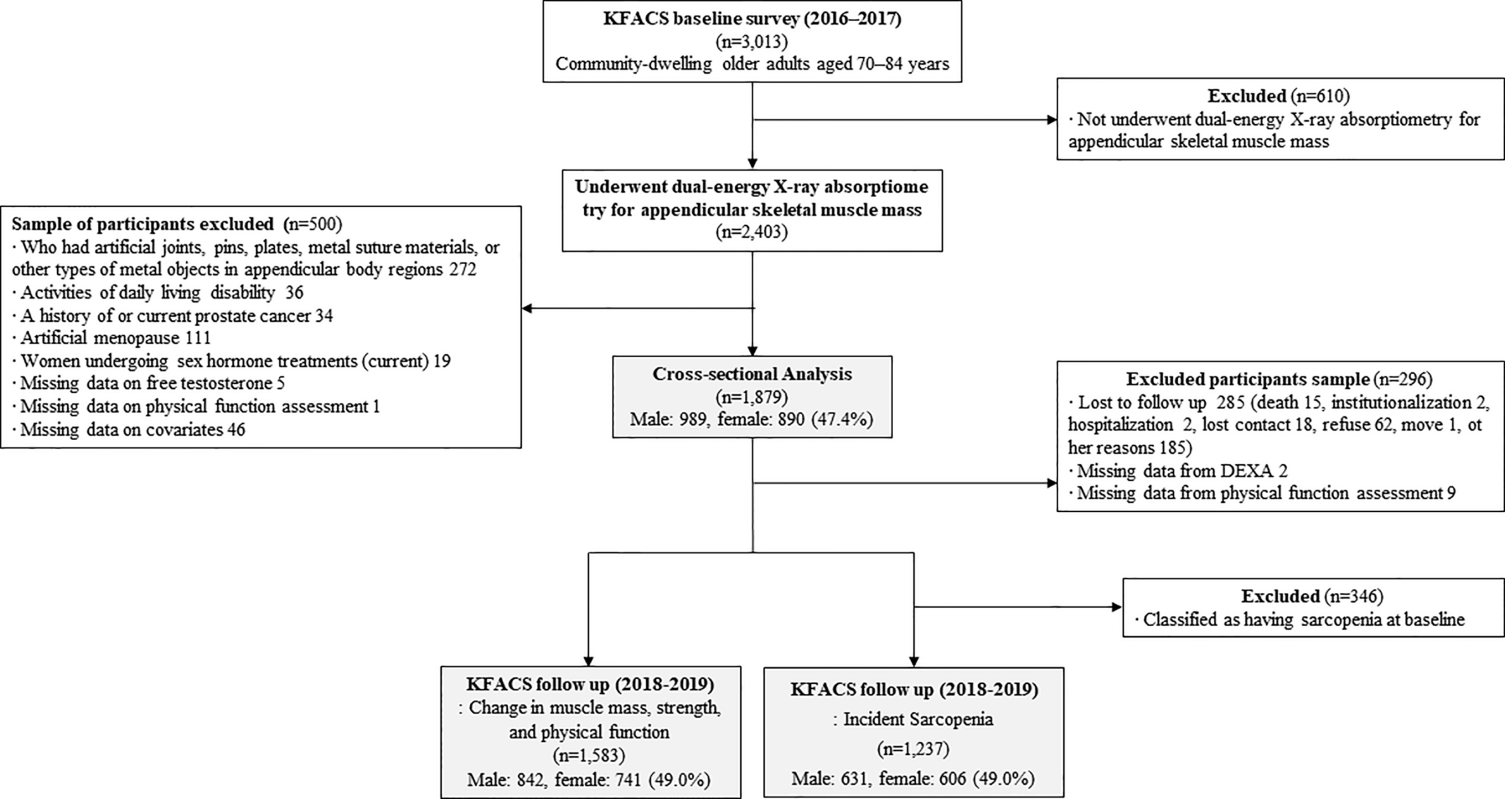
Flow chart of the study population.

The Korean Frailty and Aging Cohort Study (KFACS) is an ongoing longitudinal cohort study with a baseline survey whose purpose is to identify frailty status and frailty transitions over time in populations of older adults. Baseline survey was conducted from May 2016 to November 2017 with longitudinal visits planned for every 2 years. A total of 3,013 participants were recruited in the baseline round from sex- and age-stratified community-dwelling older adults aged 70–84 years in ten study centers, which included urban, suburban, and rural areas throughout South Korea (26). The ratio of age was 6:5:4 for 70–74, 75–79, and 80–84 years, respectively, while that of sex was 1:1. To minimize selection bias, the participants were recruited from various settings such as local senior welfare centers, community health centers, apartments, housing complexes, and outpatient clinics. Body composition was assessed by bioelectrical impedance analysis (BIA) in two health centers (n=610) and dual-energy X-ray absorptiometry (DXA) in eight hospital centers (n=2,403). A total of 610 participants whose body composition was assessed by BIA were excluded due to the presence of systematic bias in the BIA and DXA measurements of the appendicular lean mass (27–29). The cross-sectional analysis included 1,879 participants after the exclusion of participants presented as follows. Participants who had artificial joints, pins, plates, metal suture materials, or other types of metal objects in the appendicular body regions were excluded (n=272). Those who were considered to be dependent on their caregivers when performing any of the basic activities of daily living (ADL) were excluded because sarcopenia is associated with functional disability as measured using the ADL scale (n=36) (30). Men who had a history of or current prostate cancer (n=34) were excluded due to the possibility of previous androgen deprivation therapy which significantly affects the sex hormone levels (31). Women who had a history of undergoing oophorectomy or hysterectomy (n=111) were excluded since testosterone levels are known to decrease in women with surgical menopause, whereas it remains stable in women who experience natural menopause (32, 33). Participants who were currently receiving hormonal treatments (n=19) and those who had missing data on free testosterone level (n=5), physical function assessment (n=1), and covariates (n=46) were also excluded. Prospective examination of the association between baseline FT level and incidence of sarcopenia were utilized in cross sectional baseline derived analysis. A total of 1,237 participants were included in the incidence of sarcopenia analyses and 1,583 participants were included in analysis for change in muscle mass, muscle strength and physical performance after 2 years. A flow chart (**Figure 1**) details reasons for inclusion or exclusion for these analyses. The Clinical Research Ethics Committee of Kyung Hee University Hospital approved the KFACS protocol (Institutional Review Board [IRB] number: 2015-12-103). This study using KFACS dataset was exempted from institutional review board approval by the Clinical Research Ethics Committee of the Kyung Hee University Hospital (IRB number: 2020-12-073).

### 2.2 Measurement of Free Testosterone and Laboratory Parameters

Blood samples were collected from the antecubital vein between 0730 h and 0800 h after an overnight fasting. Participants were required to not take medications prior to blood collection. The blood samples collected in serum separator tubes were clotted at room temperature for 30–60 min. After separation at 3,000 rpm for 10 min, blood samples were immediately stored at 2–8°C and transferred to the central clinical laboratories (Seegene Medical Foundation, Seoul, Korea) within 24 h. All serum samples were stored at -80°C prior to analysis. Serum FT levels were measured at baseline. FT levels were measured using radioimmunoassay (AMP 10-R4100), with a detection limit of 0.40 pg/mL. The inter-assay coefficients of variation (CVs) were 9.3%, 5.7%, and 11,7%, and the intra-assay CVs were 19.5%, 7.3%, and 9.1% in the low, medium, and high pools, respectively. Conventional units of FT concentrations (pg/mL) were multiplied by 3.47 to convert to SI units (pmol/L). The other hematological, biochemical, metabolic, and inflammatory parameters were as follows: serum albumin (colorimetric assay, Tina-quant Albumin Gen.2, Roche ALBT2), creatinine (kinetic colorimetric assay, creatinine Jaffé Gen.2, Roche CREJ2), hemoglobin (flow cytometry, Cellpack™ DCL, DCL-300A, Sysmex), glycosylated hemoglobin (high performance liquid chromatography assay, HLC-873G8, Tosoh), total cholesterol (enzymatic test, Cholesterol Gen.2, Roche CHOL2), triglyceride (enzymatic test, Roche TRIGL), high-density lipoprotein cholesterol (homogeneous enzymatic colorimetric test, HDL-Cholesterol Gen.4, Roche), 25-hydroxy vitamin D (chemiluminescent immunoassay, B24838, Beckman coulter), and high-sensitivity C-reactive protein (turbidimetric immunoassay, Roche). Homeostatic model assessment of insulin resistance (HOMA-IR) was calculated using the following formula: [fasting insulin (µU/mL) × fasting glucose (mmol/L)]/22.5.

### 2.3 Definition of Sarcopenia

The Asian Working Group for Sarcopenia (AWGS) defines sarcopenia as low muscle mass, low muscle strength, and/or low physical performance (34). The appendicular skeletal muscle mass (ASM) was measured using DXA (Hologic DXA; Hologic Inc., Bedford, MA, USA) and Lunar (GE Healthcare, Madison, WI, USA), and was calculated as the sum of the lean mass in both arms and legs (kg). The cutoff for low muscle mass in the diagnosis of sarcopenia was an ASM index (ASM/height2) of <7.0 kg/m2 in men and <5.4 kg/m2 in women. The handgrip strength of each hand was measured twice using a digital handgrip dynamometer (T.K.K.5401; Takei Scientific Instruments Co., Ltd., Tokyo, Japan), and the maximal value was used. We used the maximal handgrip strength of four measurements in total (two per each hand) as an output. Low muscle strength was defined as a handgrip strength of <28 kg in men and <18 kg in women. The usual gait speed was measured over a 4-m course with acceleration and deceleration phases of 1.5 m using an automatic timer (Gaitspeedmeter, Dynamicphysiology, Daejeon, Korea). The test was repeated twice, and the average of the two trials was used. Slow gait speed was defined as a gait speed of <1.0 m/s.

### 2.4 Observed Physical Performance Tests

The Short Physical Performance Battery (SPPB) test is a validated test that assesses lower extremity function by measuring balance, gait speed, and time to rise from a chair five times (35). Participants were assigned a score of 0 to 4 for the balance test and 1 to 4 for the gait speed and chair stand. If no data were missing, the total SPPB score was calculated as the sum of the three individual scores. However, if one of the three measurements was missing, the sum of the two non-missing scores plus the mean of the two non-missing scores was used as the total score. If two or three of the individual scores were missing, the total score was set to “missing” (36). The Timed “Up & Go” (TUG) test is a validated test for evaluating functional mobility. The TUG test assesses the time taken by the participants to stand up from a chair, walk a distance of 3 m, return to the chair, and sit down (37).

### 2.5 Other Measurements

For the health examinations, all participants underwent face-to-face interviews based on standardized surveys. Sociodemographic and lifestyle information on education level, smoking status, alcohol consumption, physical activity level, and living conditions were obtained by trained investigators. Comorbidities were self-reported status diagnosed by physicians and defined as two or more of the following diseases: hypertension, diabetes mellitus, dyslipidemia, myocardial infarction, congestive heart failure, angina pectoris, cerebrovascular disease, peripheral vascular disease, osteoarthritis, rheumatoid arthritis, osteoporosis, asthma, or chronic obstructive pulmonary disease. Low physical activity level was defined as total energy consumption of <494.65 kcal for men and <283.50 kcal for women, which corresponds to the lowest 20% of the total energy consumed in the general population of Korean older adults (38). The participants were asked to rate their self-perceived health as poor, fair, good, very good, or excellent. The responses “poor” and “fair” were considered to be fair/poor self-perceived health.

### 2.6 Statistical Analyses

Baseline descriptive statistical analyses were carried out to compare the participants’ characteristics according to FT quartiles. One-way analysis of variance with Bonferroni post-hoc test for continuous variables and the chi-squared test or Fisher’s exact test for categorical variables were conducted. Cross-sectional associations between serum FT levels and the prevalence of sarcopenia measured during the baseline assessment were assessed using multivariate logistic regression. FT levels are expressed as continuous (per standard deviation [SD]) and as quartiles, with the first quartile as the reference group. We analyzed the associations of continuous FT levels and FT quartiles with sarcopenia to identify the linear and threshold effects of FT levels on sarcopenia, respectively.

The associations between serum FT levels and the incidence of sarcopenia was investigated using multivariate logistic regression. In additional analyses, generalized estimating equation (GEE) models were employed to examine the associations of low baseline serum FT as a binary variable with changes in muscle mass, muscle strength, and physical performance over 2 years. GEE models, which is an extension of the generalized linear model, consider the dependency of repeated measures within participants. For this analysis, baseline FT (continuous and dichotomous) levels, time and time interactions with baseline FT was included, and the correlation structure was set to autoregressive. In GEE models, we also presented False Discovery Rate (FDR) adjusted p-value to reduce the risk of false positive due to multiple comparisons. A low FT level was defined as the lowest quartile group.

To account for potential confounders, all models were adjusted as follows: model 1, unadjusted; model 2, adjusted for age and waist circumference (39); model 3, further adjusted for smoking status, alcohol intake (≥2–3 times/week), number of comorbidities, and low physical activity (<494.65 kcal/week for men and <283.50 kcal/week for women) (40, 41); and model 4, further adjusted for albumin, triglyceride, total cholesterol, HDL-C, hemoglobin, glycosylated hemoglobin, and log-transformed hs-CRP (41–43). All the analyses were conducted for each sex separately. The statistical significance level was set at a two-sided p-value of <0.05. Statistical analyses were performed using the SPSS software (version 25.0; IBM Corp., Armonk, NY, USA).

## 3 Results

### 3.1 Descriptive Characteristics of the Study Population

Baseline characteristics according to the FT quartiles are presented in **Table 1** (men) and 2 (women). The mean age of men in our study was 76.3 ± 3.9 years, with a mean serum FT level of 32.5 ± 11.2 pmol/L at baseline. Of the 989 men at baseline, 199 (20.1%) were classified as having sarcopenia. The proportion of men with low muscle strength or low physical performance significantly differed across the FT quartiles (p<0.05). The mean age of women in our study was 75.6 ± 3.9 years, with a mean serum FT level of 3.0 ± 1.8 pmol/L. Of the 890 women at baseline, 112 (12.6%) were classified as having sarcopenia. The proportion of women with low muscle mass was significantly different according to the FT quartiles (p<0.05). The FT levels were normally distributed in both men and women.

**Table 1:** Baseline characteristics according to the free testosterone quartiles.

	Free testosterone levels (pmol/L)												
Variable	Men						Women						
	Overalln=989	1^st^ quartile(≤25.26)n=249	2^nd^ quartile(25.26–32.10)n=250	3^rd^ quartile(32.10–39.49)n=245	4^th^ quartile(>39.49)n=245	*p*	Overalln=890	1^st^ quartile(≤1.77)n=221	2^nd^ quartile(1.77–2.81)n=224	3^rd^ quartile(2.81–3.99)n=227		4^th^ quartile(>3.99)n=218	*p*
Age (years)	76.3 ± 3.9	77.1 ± 3.8	76.3 ± 4.1	75.6 ± 3.9	75.9 ± 4.1	**0.003**	75.6 ± 3.9	75.9 ± 4.1	75.6 ± 3.9	75.2	± 3.8	75.6 ± 3.9	0.285
Education (<7 years)	206 (20.9)	59 (23.7)	45 (18.0)	473 (53.2)	145 (65.9)	0.350	473 (53.2)	145 (65.9)	127 (56.7)	104	(45.8)	97 (44.5)	**<.001**
BMI (kg/m²)	24.0 ± 2.9	24.6 ± 2.9	24.1 ± 2.8	24.4 ± 2.8	24.2 ± 3.1	**<.001**	24.4 ± 2.8	24.2 ± 3.1	24.3 ± 2.7	24.5	± 2.7	24.6 ± 2.8	0.525
Waist circumference (cm)	88.6 ± 8.4	90.8 ± 8.4	89.0 ± 8.2	85.9 ± 8.2	86.2 ± 8.4	**<.001**	85.9 ± 8.2	86.2 ± 8.4	85.5 ± 8.4	86.0	± 7.7	85.9 ± 8.4	0.826
ASM/height² (kg/m²)	7.1 ± 0.8	7.0 ± 0.8	7.1 ± 0.8	5.8 ± 0.7	5.7 ± 0.7	0.193	5.8 ± 0.7	5.7 ± 0.7	5.8 ± 0.7	5.9	± 0.7	5.8 ± 0.7	**0.019**
Current smoker	110 (11.1)	33 (13.3)	16 (6.4)	9 (1.0)	5 (2.3)	0.053	9 (1.0)	5 (2.3)	1 (0.4)	2	(0.9)	1 (0.5)	0.181
Alcohol consumption (≥2–3 times/week)	339 (34.3)	75 (30.1)	80 (32.0)	33 (3.7)	9 (4.1)	0.145	33 (3.7)	9 (4.1)	7 (3.1)	9	(4.0)	8 (3.7)	0.953
Low physical activity	94 (9.5)	25 (10.0)	21 (8.4)	91 (10.2)	28 (12.7)	0.901	91 (10.2)	28 (12.7)	22 (9.8)	22	(9.7)	19 (8.7)	0.554
Number of comorbidities													
<2	579 (58.5)	121 (48.6)	150 (60.0)	371 (41.7)	83 (37.6)	**0.003**	371 (41.7)	83 (37.6)	99 (44.2)	99 (43.6)		90 (41.3)	0.477
≥2	410 (41.5)	128 (51.4)	100 (40.0)	519 (58.3)	138 (62.4)		519 (58.3)	138 (62.4)	125 (55.8)	128 (56.4)		128 (58.7)	
Hypertension	522 (52.8)	139 (55.8)	129 (51.6)	508 (57.1)	118 (53.4)	0.736	508 (57.1)	118 (53.4)	114 (50.9)	139 (61.2)		137 (62.8)	**0.026**
Diabetes	247 (25.0)	78 (31.3)	62 (24.8)	170 (19.1)	42 (19.0)	0.042	170 (19.1)	42 (19.0)	49 (21.9)	45 (19.8)		34 (15.6)	0.403
Dyslipidemia	241 (24.4)	76 (30.5)	62 (24.8)	360 (40.4)	90 (40.7)	**0.014**	360 (40.4)	90 (40.7)	82 (36.6)	90 (39.6)		98 (45.0)	0.351
Cerebrovascular disease	57 (5.8)	20 (8.0)	11 (4.4)	27 (3.0)	8 (3.6)	0.341	27 (3.0)	8 (3.6)	8 (3.6)	6 (2.6)		5 (2.3)	0.801
Fair/poor self-perceived health	184 (18.6)	57 (22.9)	45 (18.0)	302 (34.0)	90 (40.7)	0.219	302 (34.0)	90 (40.7)	83 (37.1)	66 (29.1)		63 (29.0)	**0.017**
Albumin (g/dL)	4.4 ± 0.3	4.4 ± 0.2	4.4 ± 0.3	4.3 ± 0.3	4.3 ± 0.3	0.330	4.3 ± 0.3	4.3 ± 0.3	4.3 ± 0.3	4.4 ± 0.2		4.4 ± 0.2	0.050
Free testosterone (pmol/L)	32.5 ± 11.2	18.9 ± 5.9	28.9 ± 2.0	3.0 ± 1.8	0.8 ± 0.6	**<.001**	3.0 ± 1.8	0.8 ± 0.6	2.3 ± 0.3	3.4 ± 0.3		5.4 ± 1.3	**<.001**
Total cholesterol (mg/dL)	168.3 ± 35.0	162.9 ± 36.2	166.7 ± 36.4	181.7 ± 35.5	182.3 ± 34.4	**0.004**	181.7 ± 35.5	182.3 ± 34.4	178.9 ± 33.9	183.9 ± 37.8		181.9 ± 35.7	0.507
High-density lipoprotein cholesterol (mg/dL)	50.7 ± 14.3	49.0 ± 14.8	49.7 ± 14.5	55.0 ± 13.5	57.3 ± 14.8	**0.007**	55.0 ± 13.5	57.3 ± 14.8	54.2 ± 12.7	52.3 ± 13.3		56.2 ± 12.9	**<.001**
Triglyceride (mg/dL)	116.7 ± 64.4	119.7 ± 56.9	119.7 ± 71.0	122.1 ± 54.2	116.4 ± 51.2	0.431	122.1 ± 54.2	116.4 ± 51.2	120.6 ± 49.8	128.4 ± 61.5		122.7 ± 53.0	0.125
High-sensitivity C-reactive protein ^†^	1.4 ± 2.0	1.6 ± 2.2	1.4 ± 2.1	1.2 ± 1.8	1.1 ± 1.6	0.205	1.2 ± 1.8	1.1 ± 1.6	1.2 ± 1.3	1.3 ± 2.0		1.4 ± 2.1	0.189
Hemoglobin (g/dL)	14.2 ± 1.3	13.8 ± 1.4	14.2 ± 1.4	12.9 ± 1.1	12.7 ± 1.2	**<.001**	12.9 ± 1.1	12.7 ± 1.2	12.8 ± 1.1	12.9 ± 1.1		13.1 ± 1.0	**0.009**
Glycosylated hemoglobin (%)	6.0 ± 0.8	6.1 ± 0.9	6.0 ± 0.8	6.0 ± 0.8	6.0 ± 0.8	**0.003**	6.0 ± 0.8	6.0 ± 0.8	6.0 ± 0.8	6.0 ± 0.7		6.0 ± 0.8	0.907
HOMA-IR ^†^	2.1 ± 4.7	3.2 ± 8.9	2.0 ± 1.9	1.7 ± 1.2	1.4 ± 1.0	**<.001**	2.0 ± 1.8	2.0 ± 1.6	2.1 ± 2.6	2.1 ± 1.3		2.0 ± 1.4	0.974
Creatine (mg/dL)	0.99 ± 0.38	1.00 ± 0.31	0.97 ± 0.21	0.98 ± 0.22	1.02 ± 0.63	0.517	0.72 ± 0.16	0.72 ± 0.19	0.71 ± 0.15	0.72 ± 0.15		0.71 ± 0.14	0.867
25-hydroxy vitamin D (ng/mL)	24.8 ± 9.5	24.9 ± 9.7	24.8 ± 9.4	23.9 ± 8.5	25.4 ± 10.3	0.341	22.5 ± 10.2	23.2 ± 10.3	22.3 ± 9.7	22.7 ± 10.9		21.7 ± 9.9	0.499
**Physical performance**													
Short Physical Performance Battery score	11.2 ± 1.2	11.0 ± 1.3	11.3 ± 1.1	11.2 ± 1.2	11.3 ± 1.1	0.089	10.7 ± 1.5	10.4 ± 1.7	10.8 ± 1.5	10.8 ± 1.5		10.6 ± 1.4	**0.028**
Timed “Up & Go” (s)	10.0 ± 2.1	10.2 ± 2.2	9.7 ± 1.9	10.1 ± 2.1	9.8 ± 2.0	**0.038**	10.4 ± 2.6	10.8 ± 2.7	10.2 ± 2.5	10.5 ± 2.7		10.2 ± 2.4	**0.043**
Hand grip strength (kg)	32.5 ± 5.6	31.7 ± 5.6	32.6 ± 5.2	33.3 ± 6.0	32.6 ± 5.4	**0.025**	21.3 ± 3.8	20.8 ± 3.8	21.4 ± 3.7	21.6 ± 4.0		21.3 ± 3.7	0.122
Five-times sit-to-stand (s) ^†^	10.5 ± 3.1	10.9 ± 3.2	10.2 ± 2.9	10.5 ± 3.2	10.2 ± 2.8	**0.034**	11.8 ± 4.0	12.3 ± 4.4	11.6 ± 4.0	11.6 ± 4.2		11.8 ± 3.4	0.225
Usual Gait speed (m/s)	1.18 ± 0.26	1.15 ± 0.29	1.18 ± 0.24	1.17 ± 0.27	1.23 ± 0.23	**0.011**	1.10 ± 0.24	1.07 ± 0.23	1.12 ± 0.26	1.08 ± 0.22		1.11 ± 0.24	0.053
**AWGS-defined sarcopenia**													
Low muscle mass	469 (47.4)	122 (49.0)	113 (45.2)	106 (43.3)	128 (52.2)	0.193	242 (27.2)	75 (33.9)	63 (28.1)	46 (20.3)		58 (26.6)	**0.013**
Low muscle strength	205 (20.7)	68 (27.3)	44 (17.6)	45 (18.4)	48 (19.6)	**0.028**	165 (18.5)	49 (22.2)	40 (17.9)	40 (17.6)		36 (16.5)	0.437
Low physical performance (only slow gait speed)	214 (21.6)	70 (28.1)	51 (20.4)	60 (24.5)	33 (13.5)	**0.001**	293 (32.9)	81 (36.7)	70 (31.3)	77 (33.9)		65 (29.8)	0.435
Sarcopenia	199 (20.1)	66 (26.5)	46 (18.4)	43 (17.6)	44 (18.0)	**0.037**	112 (12.6)	39 (17.6)	25 (11.2)	24 (10.6)		24 (11.0)	0.076

Values are means (± SD) or numbers (%). The p-values were calculated by analysis of variance (ANOVA) for continuous variables and chi-square or Fisher’s exact test for categorical variables. ASM, appendicular skeletal muscle mass; BMI, body mass index; HOMA-IR, homeostasis model assessment for insulin resistance; AWGS, Asian Working Group for Sarcopenia. Comorbidities included hypertension, myocardial infarction, dyslipidemia, diabetes mellitus, congestive heart failure, angina pectoris, peripheral vascular disease, cerebrovascular disease, osteoarthritis, rheumatoid arthritis, osteoporosis, asthma, or chronic obstructive pulmonary disease, as diagnosed by a physician. p<0.05, indicated in bold. † Some data were missing.

### 3.2 Cross-Sectional Relationship Between Serum FT Levels and Prevalence of Sarcopenia Components

In men, the cross-sectional association between circulating levels of FT and sarcopenia are presented in **Table 2**. In the multivariate logistic regression analysis adjusted for confounding factors (Model 4), higher FT level was significantly associated with lower prevalence of sarcopenia in men; a unit SD increase in FT was associated with a 5% decrease in the likelihood of being sarcopenic (odds ratio [OR]=0.95; 95% confidence interval [CI]=0.89–1.00). Compared to the highest quartile group in men, the lowest quartile FT group tended to show a higher likelihood of being sarcopenic after consideration of all confounders, although the difference was not significant (p=0.079). When identifying individual components of sarcopenia, the likelihood of having low physical performance (slow gait speed) was significantly higher in the lowest quartile of FT than in the reference group (OR=1.83, p=0.018). Men with FT levels of 32.10–39.49 pmol/L were 2.20 times more likely to have low physical performance than those with FT levels of ≥39.49 pmol/L. Low muscle mass and muscle strength were not significantly associated with the FT levels.

**Table 2:** Cross-sectional associations between baseline free testosterone levels and prevalence of sarcopenia in men (n=989).

	Model 1		Model 2		Model 3		Model 4	
	OR (95% C.I.)	p-value	OR (95% C.I.)	p-value	OR (95% C.I.)	p-value	OR (95% C.I.)	p-value
**Low muscle mass**								
1^st^ quartile (≤25.26)	0.88 (0.62–1.25)	0.470	1.13 (0.77–1.66)	0.550	1.11 (0.75–1.64)	0.608	1.14 (0.77–1.71)	0.515
2^nd^ quartile (25.26–32.10)	0.75 (0.53–1.07)	0.117	0.88 (0.60–1.29)	0.507	0.89 (0.61–1.31)	0.563	0.92 (0.62–1.35)	0.665
3^rd^ quartile (32.10–39.49)	0.70 (0.49–1.00)	**0.047**	0.78 (0.53–0.13)	0.188	0.77 (0.52–1.12)	0.171	0.78 (0.53–1.15)	0.204
4^th^ quartile (>39.49)	Reference		Reference		Reference		Reference	
**Low muscle strength**								
1^st^ quartile (≤25.26)	1.54 (1.01–2.35)	**0.044**	1.43 (0.91–2.24)	0.120	1.33 (0.84–2.10)	0.226	1.29 (0.80–2.06)	0.296
2^nd^ quartile (25.26–32.10)	0.88 (0.56–1.38)	0.569	0.84 (0.52–1.35)	0.466	0.85 (0.52–1.37)	0.500	0.84 (0.52–1.37)	0.489
3^rd^ quartile (32.10–39.49)	0.92 (0.59–1.45)	0.730	0.94 (0.59–1.51)	0.800	0.91 (0.56–1.46)	0.689	0.92 (0.56–1.49)	0.721
4^th^ quartile (>39.49)	Reference		Reference		Reference		Reference	
**Low physical performance** **(only slow gait speed)**								
1^st^ quartile (≤25.26)	2.51 (1.59–3.98)	**<0.001**	2.20 (1.36–3.54)	**0.001**	1.96 (1.20–3.19)	**0.007**	1.83 (1.11–3.01)	**0.018**
2^nd^ quartile (25.26–32.10)	1.65 (1.02–2.66)	**0.041**	1.55 (0.95–2.54)	0.081	1.52 (0.92–2.51)	0.099	1.54 (0.93–2.56)	0.093
3^rd^ quartile (32.10–39.49)	2.08 (1.30–3.33)	**0.002**	2.14 (1.32–3.46)	**0.002**	2.09 (1.28–3.41)	**0.003**	2.20 (1.34–3.62)	**0.002**
4^th^ quartile (>39.49)	Reference		Reference		Reference		Reference	
**Sarcopenia**								
Continuous levels ^a^								
Free testosterone (per standard deviation increase)	0.93 (0.88–0.98)	**0.004**	0.93 (0.88–0.98)	**0.008**	0.94 (0.89–0.99)	**0.024**	0.95 (0.89–1.00)	**0.044**
Quartiles ^b^								
1^st^ quartile (≤25.26)	1.65 (1.07–2.54)	**0.023**	1.71 (1.07–2.75)	**0.025**	1.57 (0.97–2.54)	0.066	1.55 (0.95–2.53)	0.079
2^nd^ quartile (25.26–32.10)	1.03 (0.65–1.63)	0.899	1.06 (0.65–1.74)	0.805	1.09 (0.67–1.79)	0.725	1.12 (0.68–1.85)	0.655
3^rd^ quartile (32.10–39.49)	0.97 (0.61–1.55)	0.906	1.04 (0.63–1.70)	0.879	0.98 (0.59–1.62)	0.935	0.99 (0.59–1.64)	0.956
4^th^ quartile (>39.49)	Reference		Reference		Reference		Reference	

^a^Association between continuous free testosterone levels and sarcopenia; ^b^Association between free testosterone quartiles and sarcopenia.

p<0.05, indicated in bold.

Model 1: unadjusted.

Model 2: adjusted for age and waist circumference.

Model 3: further adjusted for smoking status, alcohol intake, number of comorbidities (hypertension, myocardial infarction, dyslipidemia, diabetes mellitus, congestive heart failure, angina pectoris, peripheral vascular disease, cerebrovascular disease, osteoarthritis, rheumatoid arthritis, osteoporosis, asthma, or chronic obstructive pulmonary disease, as diagnosed by a physician), and low physical activity.

Model 4: further adjusted for albumin, triglyceride, total cholesterol, high-density lipoprotein cholesterol, hemoglobin, glycosylated hemoglobin, and log-transformed high-sensitivity C-reactive protein

The results of the multivariate logistic regression analysis of the cross-sectional association between circulating levels of FT and sarcopenia in women are shown in **Table 3**. After adjusting for all potential confounders (Model 4), serum FT level was associated with the prevalence of sarcopenia. A unit SD increase in FT was associated with a 36% decrease in the likelihood of being sarcopenic. In addition, the risk for being sarcopenic in the lowest FT level group was 1.93 times higher than in the highest FT quartile after adjusting for all covariates. When we examined the individual components of sarcopenia, the likelihood of having low muscle mass was 1.64 times higher in the lowest quartile group than in the reference group. Low muscle strength and physical performance were not significantly associated with FT levels.

**Table 3:** Cross-sectional associations between baseline free testosterone levels and prevalence of sarcopenia in women (n=890).

	Model 1		Model 2		Model 3		Model 4		
	OR (95% C.I.)	p-value	OR (95% C.I.)	p-value	OR (95% C.I.)	p-value	OR (95% C.I.)		p-value
**Low muscle mass**									
1^st^ quartile (≤1.77)	1.42 (0.94–2.13)	0.095	1.48 (0.96–2.26)	0.077	1.47 (0.95–2.28)	0.081	1.64 (1.04–2.56)		**0.032**
2^nd^ quartile (1.77–2.81)	1.08 (0.71–1.64)	0.720	1.05 (0.68–1.63)	0.826	1.04 (0.67–1.62)	0.861	1.14 (0.73–1.79)		0.571
3^rd^ quartile (2.81–3.99)	0.70 (0.45–1.09)	0.115	0.70 (0.44–1.11)	0.132	0.69 (0.44–1.10)	0.123	0.73 (0.46–1.18)		0.196
4^th^ quartile (>3.99)	Reference		Reference		Reference		Reference		
**Low muscle strength**									
1^st^ quartile (≤1.77)	1.44 (0.89–2.32)	0.135	1.46 (0.90–2.35)	0.122	1.40 (0.86–2.28)	0.182	1.31 (0.79–2.17)		0.291
2^nd^ quartile (1.77–2.81)	1.10 (0.67–1.80)	0.708	1.12 (0.69–1.85)	0.642	1.09 (0.66–1.81)	0.737	1.10 (0.64–1.80)		0.783
3^rd^ quartile (2.81–3.99)	1.08 (0.66–1.77)	0.756	1.12 (0.68–1.84)	0.662	1.14 (0.69–1.89)	0.607	1.20 (0.72–2.02)		0.486
4^th^ quartile (>3.99)	Reference		Reference		Reference		Reference		
**Low physical performance** **(only slow gait speed)**									
1^st^ quartile (≤1.77)	1.36 (0.91–2.03)	0.129	1.28 (0.84–1.94)	0.252	1.32 (0.87–2.03)	0.197	1.23 (0.79–1.89)		0.359
2^nd^ quartile (1.77–2.81)	1.07 (0.71–1.60)	0.744	1.11 (0.73–1.69)	0.624	1.08 (0.70–1.66)	0.721	1.06 (0.68–1.64)		0.800
3^rd^ quartile (2.81–3.99)	1.21 (0.81–1.80)	0.353	1.32 (0.87–2.01)	0.194	1.35 (0.88–2.06)	0.170	1.33 (0.86–2.05)		0.196
4^th^ quartile (>3.99)	Reference		Reference		Reference		Reference		
**Sarcopenia**									
Continuous levels ^a^									
Free testosterone (per standard deviation increase)	0.66 (0.44–0.99)	**0.042**	0.66 (0.44–1.00)	0.052	0.68 (0.45–1.03)	0.066	0.64 (0.42–0.99)		**0.044**
Quartiles ^b^									
1^st^ quartile (≤1.77)	1.73 (1.00–2.99)	**0.049**	1.79 (1.02–3.17)	**0.044**	1.76 (0.99–3.12)	0.053	1.93 (1.07–3.47)		**0.028**
2^nd^ quartile (1.77–2.81)	1.02 (0.56–1.84)	0.960	0.99 (0.54–1.84)	0.983	0.97 (0.52–1.80)	0.916	1.08 (0.58–2.03)		0.805
3^rd^ quartile (2.81–3.99)	0.96 (0.53–1.74)	0.882	1.05 (0.57–1.95)	0.870	1.04 (0.56–1.93)	0.914	1.12 (0.59–2.10)		0.729
4^th^ quartile (>3.99)	Reference		Reference		Reference		Reference		

^a^Association between continuous free testosterone level and sarcopenia; ^b^Association between free testosterone quartiles and sarcopenia.

p<0.05, indicated in bold.

Model 1: unadjusted.

Model 2: adjusted for age and waist circumference.

Model 3: further adjusted for smoking status, alcohol intake, number of comorbidities (hypertension, myocardial infarction, dyslipidemia, diabetes mellitus, congestive heart failure, angina pectoris, peripheral vascular disease, cerebrovascular disease, osteoarthritis, rheumatoid arthritis, osteoporosis, asthma, or chronic obstructive pulmonary disease, as diagnosed by a physician), and low physical activity.

Model 4: further adjusted for albumin, triglyceride, total cholesterol, high-density lipoprotein cholesterol, hemoglobin, glycosylated hemoglobin, and log-transformed high-sensitivity C-reactive protein

### 3.3 Longitudinal Relationships Between Serum FT Levels and Sarcopenia Components

#### 3.3.1 Changes in Muscle Mass, Muscle Strength, and Physical Performance

The associations of baseline FT levels with changes in muscle mass, muscle strength, and physical performance over 2 years are demonstrated in **Table 4**. FT levels were not associated with change in ASM index in both men and women. In men, higher FT levels were associated with more decline in time for TUG test (p=0.011, FDR-adjusted p=0.022). In women, lowest FT quartile was associated with a greater decrease in handgrip strength (p=0.010, FDR-adjusted p=0.040), though FT levels were not associated with handgrip strength (p=0.088, FDR-adjusted p=0.176).

**Table 4:** Associations of baseline free testosterone with changes in muscle mass, muscle strength, and physical performance over 2 years.

Changes in muscle mass, muscle strength, and physical performance	Men (n=842)									Women (n=741)							
	FT (continuous) × time				Lowest FT quartile ^a^ × time					FT (continuous) × time				Lowest FT quartile ^b^ × time			
	β	(95% CI)	p-value	p^*^		β	(95% CI)	p-value	p^*^	β	(95% CI)	p-value	p^*^	β	(95% CI)	p-value	p^*^
ASM index (kg/m²)	0.01	(-0.00, 0.02)	0.097	0.388		-0.04	(-0.12, 0.05)	0.385	0.513	0.01	(-0.06, 0.07)	0.877	0.877	0.05	(-0.04, 0.13)	0.292	0.513
Usual gait speed (m/s)	-0.00	(-0.01, 0.00)	0.297	0.594		-0.01	(-0.05, 0.03)	0.621	0.621	0.01	(-0.02, 0.03)	0.536	0.621	-0.03	(-0.06, 0.01)	0.121	0.484
Grip strength (kg)	0.05	(-0.03, 0.12)	0.205	0.273		-0.29	(-0.84, 0.27)	0.311	0.311	0.34	(-0.05, 0.72)	0.088	0.176	-0.61	(-1.07, -0.15)	**0.010**	**0.040**
Timed “Up and Go” test (s)	-0.06	(-0.11, -0.01)	**0.011**	**0.022**		0.53	(0.14, 0.92)	**0.008**	**0.022**	-0.00	(-0.36, 0.35)	0.985	0.985	-0.04	(-0.50, 0.42)	0.858	0.985
Short Physical Performance Battery score	0.03	(-0.00, 0.05)	0.055	0.120		-0.20	(-0.40, 0.01)	0.060	0.120	0.07	(-0.14, 0.27)	0.538	0.717	0.04	(-0.23, 0.31)	0.773	0.773
Five-times sit-to-stand (s)	-0.02	(-0.08, 0.04)	0.554	0.908		0.03	(-0.47, 0.53)	0.908	0.908	-0.35	(-0.86, 0.16)	0.177	0.708	0.05	(-0.61, 0.71)	0.885	0.908

a reference: lowest quartile (≤ 25.26pmol/L)

b reference: lowest quartile (≤ 1.77pmol/L)

^*^FDR adjusted p-value

Baseline FT × time (wave 2) for the interaction between baseline FT and muscle mass, muscle strength, and physical performance over time. Generalized estimating equation (GEE) models were used to examine the associations of low baseline FT with changes in muscle mass, muscle strength, and physical performance over 2 years. All models were adjusted for age, waist circumference, smoking status, alcohol intake, number of comorbidities (hypertension, myocardial infarction, dyslipidemia, diabetes mellitus, congestive heart failure, angina pectoris, peripheral vascular disease, cerebrovascular disease, osteoarthritis, rheumatoid arthritis, osteoporosis, asthma, or chronic obstructive pulmonary disease, as diagnosed by a physician), low physical activity, albumin, triglyceride, total cholesterol, high-density lipoprotein cholesterol, hemoglobin, glycosylated hemoglobin, and log-transformed high-sensitivity C-reactive protein. Lowest quartile was defined as ≤25.26 pmol/L in men and FT ≤ 1.77 pmol/L in women. ASM, appendicular skeletal muscle mass; CI, confidence interval; FT, free testosterone. p<0.05, indicated in bold.

#### 3.3.2 Incidence of Sarcopenia

Of the 1,237 participants classified as non-sarcopenia at baseline, the incidence of sarcopenia was 11.1% (n=70) in men and 9.7% (n=59) in women after 2 years. **Table 5** demonstrates the association between serum FT level and the incidence of sarcopenia using multivariate logistic regression. In the unadjusted and adjusted model, there were no significant associations between FT quartiles and the incidence of sarcopenia.

**Table 5:** Odds ratio (OR) for incidence of sarcopenia according to the baseline free testosterone quartiles after 2-year follow-up period.

	Model 1			Model 2			Model 3			Model 4		
	OR (95% C.I.)		p-value	OR (95% C.I.)		p-value	OR (95% C.I.)		p-value	OR (95% C.I.)		p-value
**Men (n=631)**												
**Free testosterone**												
Per standard deviation increase, continuous	0.96	(0.89–1.04)	0.297	0.96	(0.89–1.04)	0.345	0.98	(0.90–1.06)	0.573	0.98	(0.89–1.07)	0.604
1^st^ quartile (≤25.26)	1.48	(0.75–2.93)	0.258	1.53	(0.75–3.12)	0.247	1.32	(0.63–2.75)	0.457	1.32	(0.62–2.81)	0.468
2^nd^ quartile (25.26–32.10)	1.05	(0.51–2.17)	0.887	1.12	(0.54–2.35)	0.761	1.08	(0.51–2.31)	0.836	1.05	(0.49–2.25)	0.911
3^rd^ quartile (32.10–39.49)	0.93	(0.45–1.91)	0.840	1.03	(0.50-2.15)	0.932	0.99	(0.47–2.09)	0.985	1.00	(0.47–2.13)	0.998
4^th^ quartile (>39.49)		Reference			Reference			Reference			Reference	
**Women (n=606)**												
**Free testosterone**												
Per standard deviation increase, continuous	0.66	(0.38–1.14)	0.137	0.63	(0.35–1.15)	0.132	0.70	(0.39–1.26)	0.236	0.74	(0.41–1.36)	0.339
1^st^ quartile (≤1.77)	1.33	(0.61–2.91)	0.473	1.44	(0.64–3.26)	0.381	1.29	(0.63–2.55)	0.549	1.15	(0.49–2.74)	0.749
2^nd^ quartile (1.77–2.81)	1.09	(0.50–2.41)	0.827	1.01	(0.44-2.30)	0.977	1.01	(0.61–2.38)	0.975	0.95	(0.41–2.23)	0.905
3^rd^ quartile (2.81–3.99)	1.35	(0.63–2.87)	0.444	1.45	(0.66–3.19)	0.358	1.50	(0.60–2.37)	0.326	1.44	(0.63–3.27)	0.384
4^th^ quartile (>3.99)		Reference			Reference			Reference			Reference	

p<0.05, indicated in bold.

Model 1: unadjusted.

Model 2: adjusted for age and waist circumference.

Model 3: further adjusted for smoking status, alcohol intake, number of comorbidities (hypertension, myocardial infarction, dyslipidemia, diabetes mellitus, congestive heart failure, angina pectoris, peripheral vascular disease, cerebrovascular disease, osteoarthritis, rheumatoid arthritis, osteoporosis, asthma, or chronic obstructive pulmonary disease, as diagnosed by a physician), and low physical activity.

Model 4: further adjusted for albumin, triglyceride, total cholesterol, high-density lipoprotein cholesterol, hemoglobin, glycosylated hemoglobin, and log-transformed high-sensitivity C-reactive protein.

## 4 Discussion

In this prospective cohort study of community-dwelling older adults aged over 70 years, low baseline FT levels were associated with a decrease in handgrip strength in women and a decline in the TUG test in men after a follow-up period of 2 years. To the best of our knowledge, this is the first prospective study that identified sex-specific differences in the association of baseline FT levels with changes in sarcopenia components in older adults using longitudinal analyses.

In women, we found a relationship between low FT levels and a decrease in muscle strength despite a short follow-up period. A previous study reported that low FT levels accelerated the risk of sarcopenia in Japanese women aged 40–79 years on follow-up after an 8.3-year period; however, the only criterion for sarcopenia was low muscle mass, and the study did not evaluate muscle strength and physical performance (16). In our longitudinal analyses, muscle strength decreased only in the lowest quartile and not proportionally with the FT levels in women. Therefore, the relationship of FT levels with muscle strength may have a threshold and may not be a linear dose-response one in women. High doses of testosterone have been shown to be associated with muscle strength, and consequently, can improve physical performance as testosterone activates satellite cell recruitment in the high doses (44). Based on the results of these studies, it can be hypothesized that the changes in muscle strength may require overcoming a certain threshold in FT levels. In addition, we confirmed the association of FT level with low muscle mass in our cross-sectional analysis, which may have affected the decline in muscle strength after 2 years because a decrease in muscle strength is likely to be accompanied by muscle mass loss (45, 46). This can be supported by the previous study reporting a decline in FT levels leads to a decrease in muscle mass, which can accelerate a reduction in muscle strength and further reduce physical function (47).

In men, our study showed low baseline FT levels were significantly associated with a decline in physical performance, but not with muscle mass and muscle strength after 2 years. In previous studies, low FT was associated with a decline in muscle mass and physical performance in men after a long follow-up of about 7 years (17, 19). In contrary, low FT levels were not related to neither a decline in muscle strength nor in physical performance in men after 3-year follow-up period in the Longitudinal Ageing Study Amsterdam and Health, Ageing, and Body Composition study (48) as well as European Male Ageing Study (EMAS) (49). In our study, an association of FT levels with a decline in physical performance in men was confirmed despite a short follow-up period. The results in our study showed that low FT levels can affect physical performance independently of muscle mass in men. It can be expected to change in muscle mass and muscle strength if follow-up period is much longer. One explanation of the discrepancy with EMAS study is that the participants in our study were older (mean age: 60 *vs* 76.3). Another differences in results may be partially attributable to assay methodology. FT level was calculated by Vermeulen method in LASA, Health ABC, and EMAS study; however, we directly estimated FT level through radioimmunoassay method, which is more accurate. Meanwhile, the lack of association of low FT levels with muscle mass loss is consistent with previous prospective study. Gielen, E. et al. (49) reported that low levels of testosterone do not predict muscle mass loss in community-dwelling middle-aged and older men. What we have in common with previous study (49) is that most participants had testosterone levels within the normal range. According to the Endocrine Society Clinical Practice Guidelines, an FT level of <17.35 pmol/L has been suggested to be indicative of androgen deficiency in older men (50). Only 7% of participants had clinically significant low FT levels in our study.

In addition, FT level was not found to be associated with the incidence of sarcopenia after 2 years in older men and women in the present study, perhaps because the follow-up period was not long enough to affect the incidence of sarcopenia. Taken together, our results suggest that low FT levels at baseline was a predictor of decrease in muscle strength in older women and decreases in physical performance in older men after 2-year follow-up in this study. It can be speculated that FT levels in men may affect physical performance in 2 years independently of changes in muscle mass; however, FT levels in women are associated with muscle mass at baseline, which may act on muscle strength in 2 years.

The strengths of our study include the size of the cohort with a large, nationwide sample of community-dwelling Korean older adults, a reliable measurement of free testosterone levels, and the use of various potential covariates. However, our study had some limitations that should be acknowledged. The 2-year duration of observation was relatively short to evaluate the association between FT level and the incidence of sarcopenia. In addition, we performed only a single assessment of FT levels at baseline. It is possible that changes in FT levels over time may lead to different results.

To conclude, low FT levels at baseline were found to be associated with a decrease in handgrip strength in women and with a decline in the TUG test in men after 2 years. The mechanisms underlying sex-specific differences in the relationship between FT levels and sarcopenia components are still not clear; therefore, further studies are needed to explore the biological pathway of FT in both sexes.

## Data Availability Statement

The data are not publicly available due to privacy or ethical restrictions. Requests to access the datasets should be directed to CW/chunwon62@naver.com.

## Author Contributions

Conceptualization, CW and MK. Methodology, CW, MK, and JW. Formal analysis, HS. Investigation and data curation, CW, MK, and HS. Writing – original drafpt preparation, HS. Writing – review and editing, CW, MK, and JW. Supervision, CW, MK, and JW. Project administration, CW. All authors contributed to the article and approved the submitted version.

## Funding

This research was supported by a grant from the Korea Health Technology R&D Project through the Korean Health Industry Development Institute (KHIDI), which was funded by the Ministry of Health and Welfare, Republic of Korea (grant number: HI15C3153, HI19C0481, HC20C0157).

## Conflict of Interest

The authors declare that the research was conducted in the absence of any commercial or financial relationships that could be construed as a potential conflict of interest.

## Publisher’s Note

All claims expressed in this article are solely those of the authors and do not necessarily represent those of their affiliated organizations, or those of the publisher, the editors and the reviewers. Any product that may be evaluated in this article, or claim that may be made by its manufacturer, is not guaranteed or endorsed by the publisher.

## Abbreviations

AWGS, Asian Working Group for Sarcopenia; BIA, bioelectrical impedance analysis; CI, confidence interval; DXA, dual-energy X-ray absorptiometry; EMAS, European Male Aging Study; FT, free testosterone; KFACS, Korean Frailty and Aging Cohort Study; OR, odds ratio; SD, standard deviation; SHBG, sex hormone-binding globulin; TUG, Timed “Up and Go”.
