# Corrigendum: Sex-Specific Differences in the Effect of Free Testosterone on Sarcopenia Components in Older Adults

**DOI:** 10.3389/fendo.2022.876640

**Published:** 2022-03-04

**Authors:** Hyung Eun Shin, Jeremy D. Walston, Miji Kim, Chang Won Won

**Affiliations:** ^1^ Department of Biomedical Science and Technology, Graduate School, Kyung Hee University, Seoul, South Korea; ^2^ Division of Geriatric Medicine and Gerontology, Department of Medicine, School of Medicine, Johns Hopkins University, Baltimore, MD, United States; ^3^ Elderly Frailty Research Center, Department of Family Medicine, College of Medicine, Kyung Hee University, Seoul, South Korea; ^4^ Department of Biomedical Science and Technology, College of Medicine, East-West Medical Research Institute, Kyung Hee University, Seoul, South Korea

**Keywords:** free testosterone, sarcopenia, community-dwelling older adults, sex-specific difference, aging

In the original article as published, the titles of **Tables 1** and **2** were incorrect.

The correct title for **Table 1** is “Baseline characteristics according to the free testosterone quartiles”, not “Characteristics of patients at baseline” as published originally.

The correct title for **Table 2** is “Cross-sectional associations between baseline free testosterone levels and prevalence of sarcopenia in men (n=989)”, not “Characteristics of patients with EGFR-TKIs resistance who received immunotherapy or chemotherapy” as published originally.

The authors apologize for this error and state that this does not change the scientific conclusions of the article in any way. The original article has been updated.

## Publisher’s Note

All claims expressed in this article are solely those of the authors and do not necessarily represent those of their affiliated organizations, or those of the publisher, the editors and the reviewers. Any product that may be evaluated in this article, or claim that may be made by its manufacturer, is not guaranteed or endorsed by the publisher.

